# The Impact of Tezepelumab in Uncontrolled Severe Asthma: A Systematic Review of Randomized Controlled Trials

**DOI:** 10.7759/cureus.32156

**Published:** 2022-12-03

**Authors:** Pooja Roy, Zahin Islam Rafa, Sharar Naiarin Haque, Tasniem Tasha, Soumyadipto B Arko, Harshita Agrawal, Md Ibrahim Razu, Anusha Parisapogu, Sadia Maisha, Mohammad A Siddique, Farhana Karim Abbasi, Nishat Shama, Supti Dev Nath, Ammy S Ghosh, Fahmina Quader

**Affiliations:** 1 Internal Medicine, Harlem Hospital Center, New York, USA; 2 Internal Medicine, Ibn Sina Medical College Hospital, Dhaka, BGD; 3 Internal Medicine, Dhaka Medical College Hospital, Dhaka, BGD; 4 Internal Medicine, Rajshahi Medical College, Rajshahi, BGD; 5 Medical Intern, Dhaka Medical College Hospital, Dhaka, BGD; 6 Department of Laboratory Medicine and Pathology, Mayo Clinic, Rochester, USA; 7 Otolaryngology, University of Iowa Hospitals and Clinics, Iowa City, USA; 8 Infectious Diseases, Mayo Clinic , Rochester, USA; 9 Internal Medicine, Sher-E-Bangla Medical College, Barisal, Barisal, BGD; 10 Internal Medicine, North East Medical College Hospital, Sylhet, BGD; 11 Internal Medicine, Community Based Medical College Bangladesh, Mymensingh, BGD; 12 Internal Medicine, Bangladesh Institute of Research and Rehabilitation in Diabetes, Endocrine and Metabolic Disorders, Dhaka, BGD; 13 Critical Care Medicine, Johns Hopkins University, Maryland, USA; 14 Internal Medicine, Institute of Applied Health Sciences, Chittagong, BGD; 15 Internal Medicine, Sir Salimullah Medical College, Dhaka, BGD

**Keywords:** feno, eosinophil, thymic stromal lymphopoietin, chronic respiratory inflammation, tezepelumab, asthma

## Abstract

Asthma, a chronic illness, is characterized by inflammation and airway constriction. Uncontrolled severe asthma is related to poor quality of life and increased utilization of health resources. Conventional treatments are associated with a significant amount of adverse effects. Recent years have seen the identification of various molecular effectors and signaling pathways as interesting targets for the biological therapy of severe asthma that is resistant to current therapies. Because they only target some downstream components of the inflammatory response in asthma, leaving other components unaffected, current biologic treatments only lower the exacerbation rate by 50%. If we focus on the upstream mediators of the inflammatory response in asthma, it might have a greater effect and be more efficient. Tezepelumab is a human monoclonal IgG2 antibody that specifically binds to thymic stromal lymphopoietin (TSLP) at the level of its TSLPR (thymic stromal lymphopoietin receptor) binding site, inhibiting the interaction between human TSLP and TSLPR. It is being used to treat the cytokines on the respiratory epithelial layer known as "alarmins." It is the only biologic drug available for treating severe uncontrolled asthma, despite limitations in biomarker and phenotype. In light of recent developments, the lack of knowledge on tezepelumab prompts us to publish a comprehensive systematic review. We discovered that regardless of blood eosinophil level and fractional exhaled nitric oxide levels, tezepelumab dramatically lowers asthma exacerbation in patients with severe uncontrolled asthma when compared to placebo. Tezepelumab also lessens patients' demand for healthcare resources while improving clinical indicators of lung function, health-related quality of life, and asthma management in patients. Tezepelumab plays a role in enhancing pre-bronchodilator FEV1 and lowering blood eosinophil count and fractional exhaled nitric oxide in patients with or without chronic allergies (FeNO). There have been no reports of fatalities or severe adverse events connected to tezepelumab.

## Introduction and background

Asthma, a chronic respiratory disorder, affects the respiratory tract that carries ventilation throughout the lung [1]. Asthma is one of the leading causes of a lack of good quality of life, debility, and increased utilization of health resources [2]. Approximately 315 million people worldwide are affected by asthma [2,3]; among them, 70% fall under the moderate-to-severe category (steps 3 to 5 of the Global Initiative for Asthma [GINA] [3]). Statistics reflect that 1.8 million people are hospitalized due to asthma each year in the United States, and around 7.5% of the total adult population is affected by it [4].

Frequently, asthma is triggered by immunoglobulin E (IgE)-associated sensitization to a wide array of environmental allergens (dust, smoke, pollen, cold air, etc.) [4]. These peptides are presented to T-cell receptors on naive T cells by MHC (major histocompatibility complex) class II, which induces differentiation into Th2 type T cells. These Th2 cells release interleukin (IL)-3, IL-4, IL-5, IL-9, IL-13, and GM-CSF (granulocyte-macrophage colony-stimulating factor), which causes mast cell activation and an IgE-associated eosinophilic response [4]. The quiescent dendritic cells transform to express a range of co-stimulatory molecules and cell adhesions when they interact with asthma allergens; these molecules facilitate the interaction of naive T cells with the dendritic cells [4].

While some allergen-specific Th2 cells start the B cell follicle's process of switching IgM to IgE, others move to the airway mucosa as a result of chemoattractants to trigger the traditional type 2 inflammatory response [4]. Preformed IgE interacts with mast cells to activate and release various pro-inflammatory chemicals, such as histamine, prostaglandin D2, and leukotriene C4, which leads to early type bronchoconstrictor response and late-phase response [4]. The former response is caused by infiltration and activation of leukocytes (eosinophils), inducing increased leukotriene C4 (LTC4) generation and airway responsiveness and remodeling [4].

A low, medium, or high dose of inhaled corticosteroids (ICSs), short-acting beta agonists, long-acting beta agonists (LABAs), long-acting muscarinic antagonists, and leukotriene receptor antagonists (LTRAs) are some of the medications currently used to treat asthma [5]. A short-acting beta-2 agonist "rescue" inhaler is usually provided to individuals diagnosed with asthma. The two inhaled LABAs that are most frequently used are formoterol and salmeterol. Despite having a significantly longer half-life than short-acting beta-2 agonists, these medications work by a similar mechanism of action, resulting in a slower start and a longer duration of impact [6,7].

Anticholinergic drugs are categorized similarly to beta-2 agonists as either having a short-acting or long-acting duration of action. Ipratropium bromide is a typical shorter-acting medicine, whereas tiotropium, aclidinium, glycopyrronium, and umeclidinium are examples of longer-acting anticholinergic medications [8,9]. Beclomethasone, budesonide nebules, flunisolide, fluticasone, and mometasone are inhaled respiratory corticosteroids typically used once or twice daily [10]. LTRAs such as Montelukast and Zafirlukast are examples of such antagonists. Another option is methylxanthine medication called theophylline; it works in two different ways, reducing the production and release of inflammatory mediators such as leukotriene and TNF-alpha [11], while Zileuton and Cromolyn stabilize mast cells and reduce the release of proinflammatory cytokines such as histamine. In circumstances where individuals do not react well to inhaled glucocorticoid treatment and are discovered to have eosinophilia, newer class monoclonal antibody immune-modulating medicines may be employed [12].

Tezepelumab, a monoclonal IgG2 antibody that is 100% human, specifically binds to the TSLPR (thymic stromal lymphopoietin receptor) binding site on human TSLP (thymic stromal lymphopoietin), blocking the interaction between human TSLP and TSLPR [13]. Additionally, tezepelumab-treated individuals showed substantially reduced blood/sputum eosinophils and fractional exhaled nitric oxide (FeNO) levels [13]. The medication had no effect on the total serum IgE levels [14].

## Review

Research question 

The question of this systematic review was to assess if the outcome and effectiveness of the latest monoclonal antibody tezepelumab will open a new horizon in managing uncontrolled asthma.

Information sources and keyword search

The following keywords were used in PubMed searches to find relevant articles: “uncontrolled asthma,” “asthma and tezepelumab,” “uncontrolled asthma and monoclonal antibody,” and “severe asthma and monoclonal antibody,” In addition, the reference lists of reports identified by this search strategy were also searched to select relevant articles. 

Inclusion and exclusion criteria

**Figure 1:**
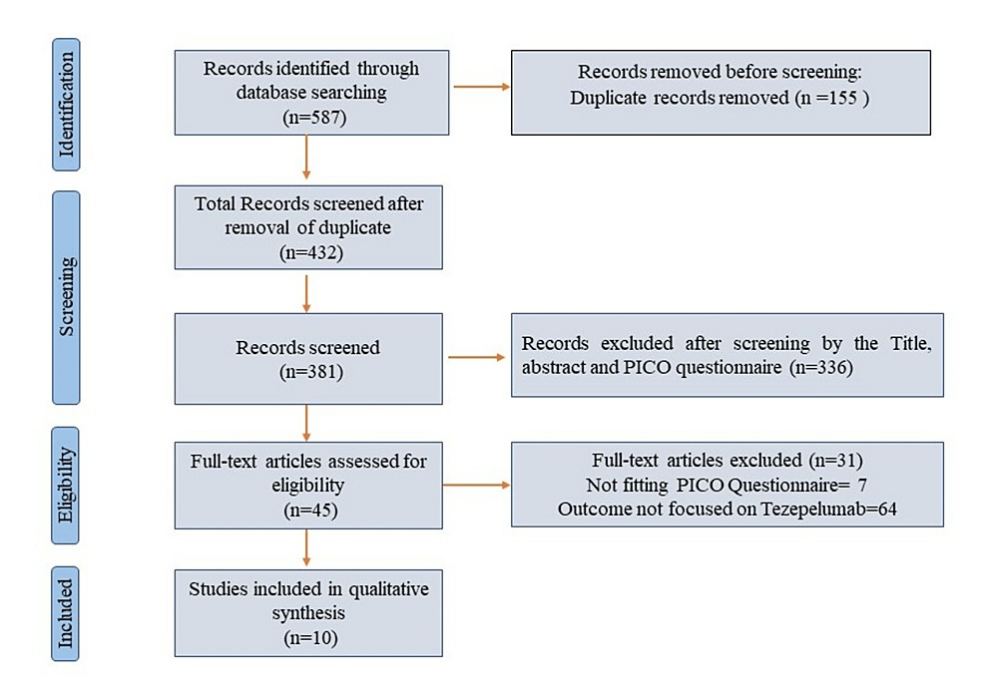
PRISMA flow diagram for the identification of the randomized controlled trials (RCTs) included in the systematic review to see the effect of tezepelumab in the treatment of asthma. PICO, Patient problem, Intervention, Comparison, and Outcome; PRISMA, Preferred Reporting Items for Systematic Reviews and Meta-Analysis

Using the search term and mesh terms, related articles were selected and reviewed. Articles were excluded not meeting the inclusion criteria and the related literature reviewed the titles and abstracts and was further assessed by examining the full texts (Figure 1).

Inclusion criteria were selected as (1) randomized controlled trials (RCTs) focusing on the effect of tezepelumab on asthma, (2) articles meeting PICO (Patient or problem, Intervention or exposure, Comparison or control) criteria, (3) established diagnosis with asthma (asthma is a chronic [long-term] condition that affects the airways in the lungs), (4) articles published in English, and (5) articles published no less than 17 years ago.

Exclusion criteria were selected as (1) case report/case series, (2) book chapter/systematic review/review, (3) editorial, and (4) articles published on animal trials, any ongoing trials.

Data collection and extraction

An initial search on PubMed for RCTs including “uncontrolled asthma”, “asthma and tezepelumab”, “uncontrolled asthma and monoclonal antibody”, and “severe asthma and monoclonal antibody” found 284, 16, 76, and 211 articles, respectively, since 2005. After the removal of the duplicates, there were 432 articles to review. After initial screening among the mentioned number, 381 articles were available; following the inclusion and exclusion criteria and the outcome of the reviewed RCTs, 45 full-text articles were compiled. After a thorough review, finally, 10 articles [15-23] were selected for table formation (Table 1).

**Table 1:** Randomized controlled trials focusing on the impact of tezepelumab in uncontrolled asthma AAER, annualized asthma exacerbation rate; ACQ-6, Asthma Control Questionnaire–6; AI, autoinjector; APFS, accessory prefilled syringe; AQLQ, Asthma Quality of Life Questionnaire; ASD, Asthma Symptom Diary; FEV1, forced expiratory volume in 1 second; HRQoL, health-related quality of life; ISRs, injection site reactions; OCS, oral corticosteroid; PK, pharmacokinetic; PRO, patient-reported outcome; SC, subcutaneous; SOURCE, Several ongoing phase 3 trials; V-S, vial and syringe

Author name and year of publication	Purpose of study	Type of study	Outcomes and results	Conclusions
Corren et al., 2021 [15]	Evaluated tezepelumab's effectiveness in treating PATHWAY individuals with chronic allergies	Phase 2b, randomized	In individuals with perennial allergies (n = 254) and in those without perennial allergies (n = 261), tezepelumab lowered the AAER against placebo by 66% to 78% and 67% to 71%, respectively. Regardless of the presence of perennial allergies, tezepelumab over a 52-week period increased prebronchodilator FEV1 and decreased blood eosinophil counts and fractional exhaled nitric oxide levels	In patients with severe, uncontrolled asthma with or without perennial allergies, tezepelumab treatment reduced exacerbations, enhanced lung function, and decreased type 2 biomarkers compared to placebo
Menzies-Gow et al., 2020 [16]	Compare the impact of tezepelumab to placebo on asthma attacks in adults and children with severe, uncontrolled asthma	Phase 3, multicenter, randomized, double-blind, placebo-controlled, parallel-group trial	Reduced asthma exacerbations, symptoms, and improved lung function	This study gave more support to the conclusions made from the earlier clinical studies with tezepelumab that it had the potential to help patients with severe, uncontrolled asthma to improve their lung function, asthma control, and, HRQoL
Menzies-Gow et Al., 2021 [17]	To investigate tezepelumab’s effectiveness and safety in people with severe, uncontrolled asthma	Phase 3, multicenter, randomized, double-blind, placebo-controlled trial	At week 52, improvements were greater with tezepelumab than with placebo with respect to the prebronchodilator FEV1 (0.23 vs. 0.09 liters; difference: 0.13 liters; 95% CI: 0.08 to 0.18; p<0.001) and scores on the ACQ-6 (−1.55 vs. −1.22; difference: −0.33; 95% CI: −0.46 to −0.20; p<0.001), AQLQ (1.49 vs. 1.15; difference, 0.34; 95% CI, 0.20 to 0.47; p<0.001), and ASD (−0.71 vs. −0.59; difference: −0.12; 95% CI: −0.19 to −0.04; p=0.002)	Compared to those who got a placebo, patients with severe, uncontrolled asthma who received tezepelumab experienced fewer exacerbations and had better lung function, asthma control, and HRQoL
Sakamoto et al., 2021 [18]	To evaluate tezepelumab's immunogenicity, PK, safety, and tolerability in healthy Japanese male volunteers	phase 1, single-center, randomized, single-blind, placebo-controlled, single-ascending dose study	In the study population of healthy Japanese men, has a favorable safety and tolerability profile.	There were no observable differences in the safety and PK of tezepelumab between Japanese and non-Japanese persons.
Wechsler et al., 2020 [19]	The major goal is to evaluate how tezepelumab compares to placebo in terms of its ability to lower the recommended OCS maintenance dose. The key secondary goal is to evaluate tezepelumab’s impact on asthma exacerbation rates. The percentage of patients obtaining an OCS dose decrease (100%, 50%, or less than 5 mg) and the impact of tezepelumab on lung function and PROs are additional secondary goals.	Phase 3, multicenter, randomized, double-blind, placebo-controlled study	Reduced asthma exacerbations, symptoms, improved lung function, and reduced OCS use	Tezepelumab's ability to spare the OCS in patients with OCS-dependent asthma is being studied by SOURCE. Additionally, SOURCE intends to show that tezepelumab therapy for people with severe asthma is linked to lower incidence of exacerbations as well as better lung function, asthma control, and HRQoL, while using less OCS.
Menzies-Gow et al., 2020 [20]	Tezepelumab is being studied for its effectiveness, safety, and ability to reduce the need for OCSs	phase 3, multicenter, randomized, double-blind, placebo-controlled, parallel-group trial	Reduced asthma exacerbations, symptoms, and improved lung function	It supports potential long-term treatment benefits in patients with severe, uncontrolled asthma
Zheng et al., 2021 [21]	The safety, tolerability, and PK properties of tezepelumab administered subcutaneously by V-S vs an AI or an APFS were examined in this study (AI).	Single-center, randomized, open-label, parallel-group study	When given through V-S, APFS, or AI, tezepelumab PK characteristics following a single 210-mg SC dosage were comparable. Injection site pain and immunogenicity rates were minimal across all groups, and ISRs were uncommon.	These findings indicate the use of APFS or AI in addition to V-S for the administration of tezepelumab, giving patients and physicians’ greater choices and possibly more convenient at-home use
Parnes et al., 2019 [22]	The study assessed tezepelumab's preliminary clinical activity in atopic dermatitis, its PK characteristics, and its initial safety in humans	Randomized, double-blind, placebo-controlled study	These studies found no evidence of immunogenicity. These outcomes are in line with the safety data from tezepelumab clinical studies in asthma patients, which did not discover a higher incidence of infection in those receiving the drug compared to those receiving a placebo.	In healthy and AD adult participants, tezepelumab was well tolerated, had a predicted linear PK profile, and had good safety and tolerability
Corren et al., 2017 [3]	In this trial, human monoclonal antibody tezepelumab was tested for its effectiveness and safety in treating patients whose asthma was uncontrolled despite receiving treatment with long-acting beta-agonists and moderate-to-high doses of inhaled glucocorticoids	Phase 2, randomized, double-blind, placebo-controlled trial	Exacerbation rates were 62%, 71%, and 66% lower in the tezepelumab groups than in the placebo group, respectively (p <0.001 for all comparisons). At week 52, all tezepelumab groups had greater prebronchodilator FEV1 than the placebo group (differences of 0.12 liters with the low dose [p=0.015], 0.13 liters with the medium dose [p=0.009], and 0.15 liters with the high dose [p=0.002])	Independent of baseline blood eosinophil levels, individuals treated with long-acting beta-agonists and medium-to-high doses of inhaled glucocorticoids who got tezepelumab had lower rates of clinically significant asthma exacerbations than those who received placebo
Corren et al., 2021 [23]	Tezepelumab reduced exacerbations by up to 71% in the phase 2b PATHWAY study (NCT02054130) compared to placebo, and it also enhanced lung function, asthma control, and health-HRQoL. The effect of tezepelumab on PROs in PATHWAY was further evaluated in this investigation.	Phase 2b, randomized, multicenter, double-blind, placebo-controlled trial	Patients in the tezepelumab-treated group had a higher percentage of well- or partially-controlled asthma than those in the placebo group. Tezepelumab also reduced the overall intensity of the symptoms.	As seen by the larger percentage of ACQ-6 and AQLQ(S)+12 responders and improvements in symptom severity in the tezepelumab dose groups, tezepelumab treatment improved PROs compared to placebo. The advantages of tezepelumab in treating patients with severe, uncontrolled asthma are further supported by these data.

Our systematic review has some limitations. Firstly, no other search tool was used apart from PubMed. To ensure high quality and prevent data extraction from fraudulent papers, we restricted it to PubMed. To our knowledge, there has been no previous systematic review focusing on the RCTs on asthma and tezepelumab carried out in humans. Our article thus can be a tool for adding tezepelumab to treat uncontrolled asthma despite the escalation of conventional management.

Discussion

Pathophysiology of Asthma

Asthma is a chronic disease of the airways that is characterized by constriction of the air passages and inflammation. When an airway is exposed to an allergen, the body responds with a type 2 inflammatory response that is characterized by the release of IL-4, IL-5, and IL-13 from antigen-specific T-helper 2 cells, the generation of IgE that causes mast cell degranulation, and the development of eosinophilia [15]. Additionally, type 2 innate lymphoid cells have been found to support the activity of immune effector cells, such as eosinophils in allergic asthma [15].

TSLP, an essential upstream cytokine also known as an alarmin, is released by airway epithelial cells in response to allergens, irritants, or infections [16-18]. Dendritic cell activation by TSLP accelerates the development of CD4 naive T cells into T-helper 2 cells by facilitating antigen presentation to CD4 naive T cells during allergic reactions [18-20]. Type 2 innate lymphoid cells respond to TSLP by producing the type 2 cytokines IL-5 and IL-13, which cause allergic reactions [15]. TSLP activates a wide variety of cell types and signaling pathways involved in type 2 and non-type 2 inflammation [15]. This includes group 2 innate lymphoid cells, mast cells, basophils, and macrophages, along with the other epithelial alarmins, IL-25, and IL-33 [18,25]. TSLP can also promote the growth of type 17 helper T cells, which aid in neutrophil recruitment [18]. Additionally, TSLP can direct the production of T2 cytokines by mast cells as well as the production of TSLP by mast cells following IgE cross-linking, all of which can promote the spread of allergic inflammation, eosinophilic inflammation, and constriction of the smooth muscles of the airways [15]. The expression of TSLP has been observed to be higher in asthma patients [18,21]. 

In patients with asthma, TSLP levels are related to airway obstruction, disease severity, and glucocorticoid resistance [17]. In addition to its fundamental role in the development of asthma, TSLP is known to play a significant role in the pathogenesis of several allergy diseases [16]. The TSLP protein is expressed abnormally in cancer and allergic gastrointestinal diseases such as Crohn's disease, eosinophilic esophagitis, and ulcerative colitis, as well as in the skin lesions of people with atopic dermatitis [16]. Similar to this, patients with chronic obstructive pulmonary disease displayed higher TSLP expression compared to healthy individuals [16].

New Horizon of Medications

Despite using regular corticosteroids, some patients still have persistent asthma symptoms [19,25-27]. Therefore, targeting specific inflammatory pathways, some biologics are developed and approved by the U.S. Food and Drug Administration (FDA) before December 2021, such as omalizumab, dupilumab, benralizumab, reslizumab, and mepolizumab [25,27,28]. In comparison to placebo reslizumab, mepolizumab and benralizumab reduced the risk of exacerbation (by 50-60%, 47-52%, and 28-51%, respectively) [27]. Benralizumab and reslizumab assist in enhancing lung function [27]. Although those biologics are only effective in treating allergic and eosinophilic asthma, patients with blood eosinophil counts (Eos) below 150 cells/L showed minimal efficacy, and those with Eos between 150 and 300 cells/L showed inconsistent efficacy [27]. We, therefore, needed a more effective biologic that could treat severe uncontrolled asthma effectively, despite limits in biomarker and phenotype (e.g., allergic or eosinophilic) [27]. Tezepelumab was given FDA approval in December 2021 [27]. Despite phenotypic and biomarker limitations, it is the only biologic for the treatment of severe uncontrolled asthma [27].

Mechanism of Action

Tezepelumab inhibits the human monoclonal antibody IgG2 that binds to TSLP, also known as TSLP. It blocks its interaction with the heterodimeric TSLP receptor [20,26]. A cytokine called TSLP, which is produced by epithelial cells, sits upstream in the inflammatory cascade that leads to asthma [20].

**Figure 2:**
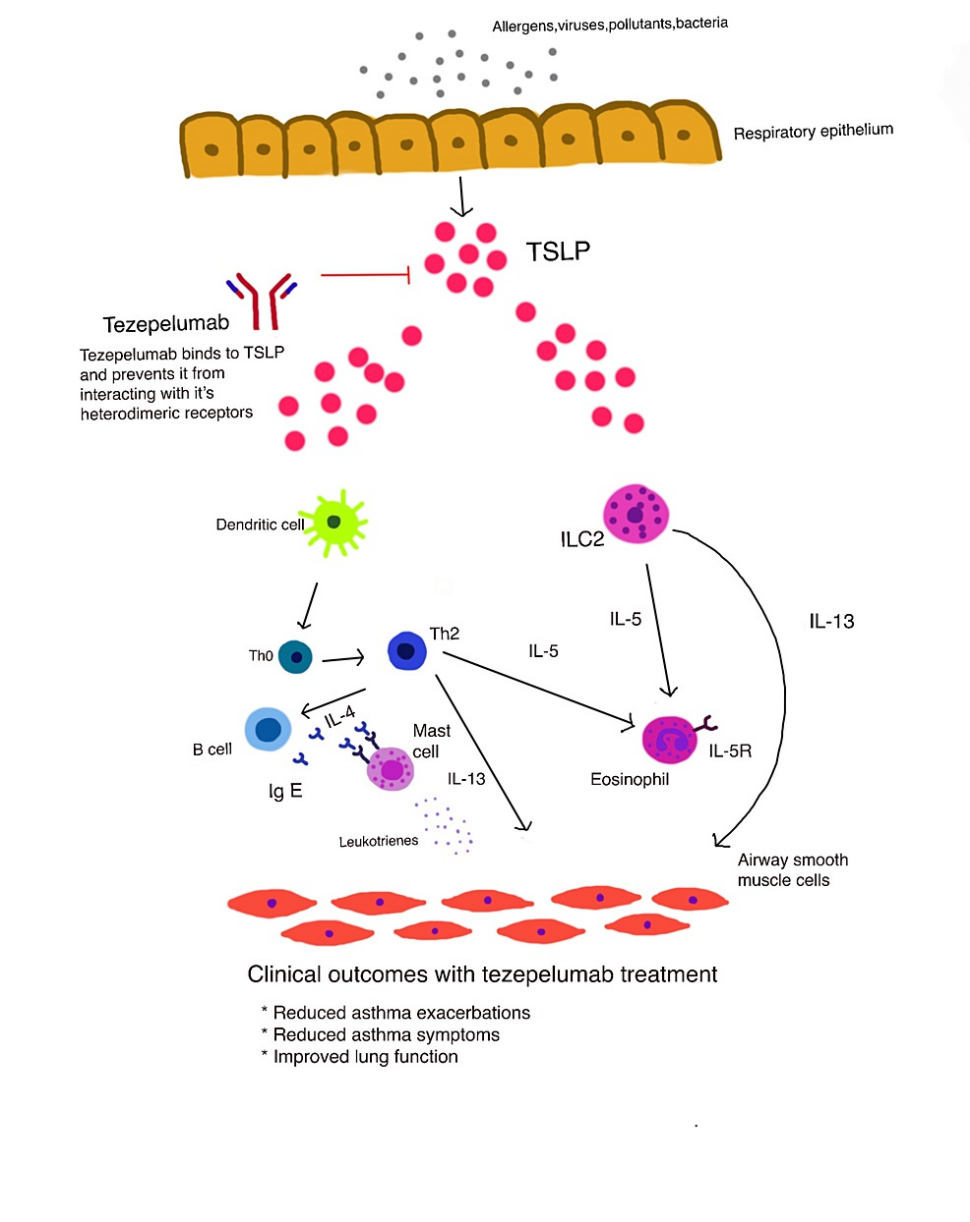
Mechanism of action of tezepelumab. This figure is a self-illustration by the authors.

The mechanism of action of tezepelumab improves the symptoms of patients with severe asthma. In response to contact with viruses, allergens, and pollutants, the airway epithelial cells release TSLP, which sets off several inflammatory cascades [20,21]. The particular binding of TSLP to its heterodimeric receptor is blocked by tezepelumab, which also inhibits the synthesis of several inflammatory cytokines and cell types. Tezepelumab treatment has been demonstrated to lower blood eosinophil count, IgE, IL-5, IL-13, and FeNO [20] (Figure 2).

Pharmacokinetics

Tezepelumab was tested in randomized, placebo-controlled research on healthy Japanese men, and the results showed a linear relationship between systemic exposure over the dose range examined when tezepelumab was delivered subcutaneously [18]. Both the SAD study and the MAD study provided more support for this linear association [22]. Tezepelumab has a slow rate of absorption and its mean Vz/F of 5,568-550 mL indicates that it has a somewhat small tissue distribution. The mean t1/2, z for tezepelumab is 23.9-26.3 days [18]. It is interesting to note that other monoclonal antibodies also have a long half-life and a restricted diffusion into peripheral organs. IgG's lengthy half-life is caused by an inhibition of lysosome degradation when it engages with the neonatal Fc receptor. Tezepelumab's mean CL/F was 147.8-175.0 mL/day and its mean Cmax was 5.2-39.7 g/mL [18]. Tezepelumab’s pharmacokinetics (PK) (Cmax, Tmax, AUC0, AUC0 last, and t12z) and serum concentration profile were identical when given a vial-and-syringe, accessorized prefilled syringe, or autoinjector (AI) through three different injection sites, according to tezepelumab PK, safety, and tolerability after administration in healthy volunteers (upper arm, abdomen, and thigh). Although there were hardly any differences between the exposure of the three device groups and the injection sites, they are considered to be clinically irrelevant. The statistical analysis showed that for all paired comparisons between vial and syringe (V-S), accessory prefilled syringe (APFS), and AI, the bioequivalence interval of 0.8-1.25 covered the 90% CI of GMRs (geometric mean ratio) for Cmax, AUC0, and AUC0 last. Researchers examined data after a single intravenous (IV) or subcutaneous (SC) tezepelumab injection for the SAD investigation and discovered that the mean half-life (t1/2, z) ranged from 19.9 to 25.7 days. Compared to the IV 210 mg dose, the SC 210 mg dose had a bioavailability of 81%, and the tmax ranged from 81 to 237 hours, or around 3 to 10 days. However, findings from the MAD research were gathered after tezepelumab was administered in numerous doses intravenously and subcutaneously. The PK parameter resembled data from a single dose. The mean (SD) areas under the concentration versus time curve across the dosing interval (AUC) accumulation ratio for the monthly (every 28 days) dose groups of 35 mg, 105 mg, and 210 mg were 1.82 (0.262), 1.64 (0.0883), and 1.59 (0.242), respectively. For the 210 mg SC dose given every 28, 14, and 7 days, the mean (SD) Cmax accumulation ratios were 1.59 (0.288), 2.84 (0.965), and 6.74 (1.69), respectively [22]. 

Adverse Effects

The overall incidence of adverse events was similar across the trial groups (Table 1) [3] Overall, 65.9% of patients in the placebo group, 67.4% in the low-dose tezepelumab group, 65.7% in the medium-dose group, and 65.0% in the high-dose group reported at least one adverse event, and 13.0%, 12.3%, 9.5%, and 13.1%, respectively, reported at least one serious adverse event [3].

The total incidence of adverse events was equivalent among the study groups [3] when asthma-related adverse events were excluded from the aforementioned analysis. The investigator determined that the trial agent was responsible for three major adverse events, two of which-pneumonia and stroke occurred in the same patient in the low-dose tezepelumab group and one with Guillain-Barré syndrome in the medium-dose tezepelumab group. Five patients, including two in the medium-dose group and three in the high-dose group, received tezepelumab at a discontinuation rate of 1.2% compared to 0.7% in the placebo group (one patient). A significant adverse event related to therapy (stroke in the case of one patient in the low-dose tezepelumab group) caused their death eight weeks after the treatment period finished.

Injections of 1 mL resulted in injection-site responses in 3.6% of patients receiving a placebo, 2.9% of patients receiving a low dose of tezepelumab, 2.9% of patients receiving a medium dose, and 1.5% of patients receiving a high dose [3]. The respective rates following 1.5 mL injections were 2.9%, 2.2%, 2.9%, and 3.6%. There were no recorded anaphylactic responses caused by experimental products. After baseline, there were 3 (2.3%) of 131 patients in the high-dose group, 3 (1.3%) of 138 patients in the medium-dose group, 5 (3.7%) of 136 patients in the low-dose tezepelumab group, and 13 (9.4%) of 138 patients in the placebo group who had positive antidrug antibodies (ADAs) [3]. There were no neutralizing antibodies found [3].

Both the tezepelumab and placebo groups experienced the same proportion of patients experiencing negative side effects. There have been no reports of fatalities or serious adverse events associated with tezepelumab in any trial subject [22]. In the MAD study, 24 (65%) of the 37 participants in the tezepelumab group and 10 (83%) of the 12 participants in the placebo group reported at least one adverse event [22]. The most common side effects were headache (22% in the tezepelumab group and 17% placebo), elevated creatine phosphokinase (8% tezepelumab, 17% placebo), viral infection (8% tezepelumab, 17% placebo), pain at the injection site (8% tezepelumab, 0% placebo), and pruritus (8% tezepelumab, 8% placebo) [26]. With tezepelumab, it appeared that the frequency of injection site reactions, such as pain, hematoma, and swelling, was higher.

Immunotolerance

According to the Randomized, Placebo-Controlled Study to Evaluate the Safety, Tolerability, Pharmacokinetics, and Immunogenicity of Subcutaneous Tezepelumab in Healthy Japanese Men study, no one who received tezepelumab produced anti-tezepelumab antibodies, indicating that the medication has low immunogenicity [18]. In agreement with this finding, the phase 2b study also showed a low incidence of anti-tezepelumab antibodies: in the dose groups receiving 70 mg (every 4 weeks), 210 mg (every 4 weeks), and 280 mg (every 2 weeks), ADAs developed in 3.7% (5/136), 0.8% (1/131), and 2.3% (3/131) of patients, respectively [18]. Tezepelumab was also found to be less immunogenic than other monoclonal antibodies used to treat asthma [18]. For instance, 37.5% (3/8), 12.5% (1/8), and 12.5% (1/8) of patients in the treatment groups developed immunogenic responses in a study of the PK and pharmacodynamics of a single dosage of mepolizumab in healthy Japanese male adults [18,19].

Tezepelumab Pharmacokinetics, Safety, and Tolerability After Administration via Vial-and-syringe, Accessorized Prefilled Syringe, or Autoinjector study showed that at 3.2% and 1.3%, respectively, the proportions of patients who tested positive for both treatment-emergent ADAs and ADAs overall in this research were modest. Tezepelumab has been discovered to have comparable low immunogenicity in earlier studies [21]. No healthy volunteer participant has ever tested positive for treatment-emergent ADA [21].

Uses

Regardless of blood eosinophil level or fractional exhaled nitric oxide levels, the phase 2b PATHWAY research demonstrates that tezepelumab effectively lowers asthma exacerbation over 52 weeks by 71% compared to placebo in individuals with severe uncontrolled asthma [23]. Tezepelumab enhances clinical measurements of lung function, asthma control, patient health-related quality of life (HRQoL), and minimal utilization of resources connected to healthcare [15,23]. When used by asthma patients who have been on oral corticosteroid (OCS) therapy for more than six months, this medication lessens exacerbation [19]. Additionally, tezepelumab lessens allergen-induced asthma exacerbations (perennial or seasonal) [17]. Tezepelumab has been shown to improve pre-bronchodilator FEV1 and decrease blood eosinophil count and FeNO in patients with or without perennial allergy, according to a post hoc investigation carried out in Los Angeles, Thousand Oaks, Gaithersburg, and Warsaw, Poland. These data support the effectiveness of tezepelumab in lowering total serum IgE in patients with perennial allergies and tezepelumab's function in TSLP inhibition for treating asthma exacerbations in patients with perennial allergies severe, uncontrolled asthma [15].

Benefits

The first biologic immunotherapy, tezepelumab, consistently and dramatically lowered exacerbations in a large cohort of individuals with severe asthma. Positive full results from the pivotal NAVIGATOR phase III trial showed that tezepelumab demonstrated a statistically significant and clinically meaningful reduction in the annualized asthma exacerbation rate (AAER) in severe, uncontrolled asthma patients [20]. Tezepelumab demonstrated superiority versus placebo across every primary and key secondary endpoint.

As added to the standard of care (SoC), the potential first-in-class drug tezepelumab reduced AAER in the total patient group by 56% (p=0.001) over 52 weeks when compared to placebo. SoC consisted of at least one additional controller drug, either with or without OCSs, as well as medium- or high-dose ICS [20]. Regardless of baseline eosinophil count, tezepelumab is the only biologic drug to consistently and significantly lower AAER in a large group of severe asthma patients across phase II and phase III clinical trials [20].

In a pre-planned subgroup analysis, tezepelumab reduced AAER in patients with baseline eosinophil counts under 300 cells per microliter by a statistically significant and clinically important 41% (p=0.001). Importantly, clinically significant decreases in AAER have also been seen in two additional subgroups: patients with baseline eosinophil counts below 150 cells per microliter saw a reduction of 39%, while patients with baseline counts above 300 cells per microliter saw a reduction of 70% [20].

In conclusion, biologic therapies have substantially advanced the treatment of severe asthma; yet, as of the writing of this article, little is understood about the genuine therapeutic benefit of monoclonal antibodies on asthma. Since it is an essential component of long-term illness management, this clinical issue should be addressed in ongoing and prospective monoclonal antibody research for the treatment of severe asthma.

**Figure 3:**
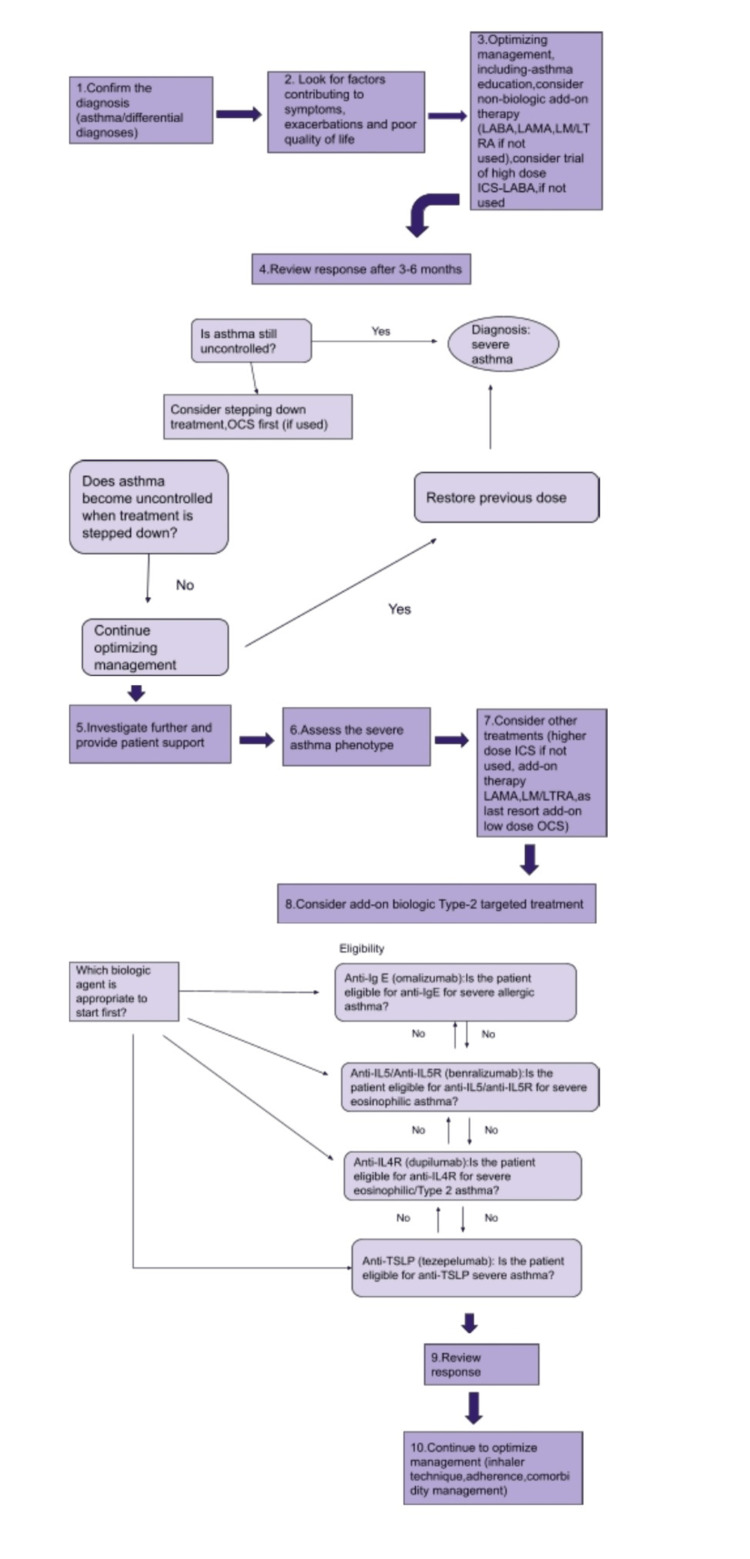
GINA Global Strategy for Asthma Management and Prevention (https://ginaasthma.org) This figure is a self-illustration by the authors. GINA, Global Initiative for Asthma

Figure 3 illustrates the Global Strategy for Asthma Management and Prevention.

## Conclusions

Asthma is a chronic debilitating lung disorder affecting almost 350 million people across the globe; among them, around 70% of people develop moderate-to-severe symptoms that are difficult to control despite following step-up asthma treatment guidelines, including long-term steroid. Tezepelumab is a new biologic drug that enlightens new treatment strategies. It shows clinically promising effects by lowering annualized asthma exacerbation, improving lung function and HRQoL, and decreasing overall health care burden in multiple significant phases III double-blinded RCTs. Tezepelumab is a monoclonal IgG2 antibody that exerts its effect by blocking the interaction between human TSLP and TSLPR and thus reduces the production of different cytokines, which is responsible for various debilitating pathophysiology of asthma. It also improves pre-bronchodilator FEV1 and decreases blood eosinophil count and FeNO. Furthermore, there were no significant adverse events compared to placebo except the higher local injection site reaction as tezepelumab was introduced by SC or IV route. All these clinically and statistically effective outcomes might encourage more studies on tezepelumab and emphasize its use in severe uncontrolled asthma.
